# Health-related quality of life in breast cancer measured with EQ-5D-5L

**DOI:** 10.1186/s41687-026-01044-x

**Published:** 2026-03-20

**Authors:** Sofia Torres, Maureen Trudeau, Geoffrey Liu, Nicholas Mitsakakis, Ahmed M. Bayoumi

**Affiliations:** 1 Institute of Health Policy, Management and Evaluation, University of Toronto, Toronto, ON 3M6 Canada; 2 Division of Medical Oncology and Malignant Hematology, Sunnybrook Health Sciences Centre, Toronto, ON M4N 3M5 Canada; 3 Division of Medical Oncology and Hematology, Princess Margaret Cancer Centre, Toronto, ON M5G 2C4 Canada; 4 Children’s Hospital of Eastern Ontario Research Institute, Ottawa, ON K1H 8L1 Canada; 5 Department of Medicine, University of Toronto, Toronto, ON M5S 3H2 Canada

**Keywords:** Breast cancer, EuroQol (EQ) 5-dimensions (5D) 5-level (5L) questionnaire, Utility, Health-related quality of life, Preferences

## Abstract

**Background:**

Health utility values are required for cost-utility analyses in breast cancer, yet EQ-5D-5L-based health utility estimates and validity evidence across clinically relevant health states remain limited. The objectives of this study were to estimate health utility values for pre-defined breast cancer health states using the EQ-5D-5L (EuroQoL 5-level) instrument and to investigate its construct validity in breast cancer.

**Methods:**

This cross-sectional study included women with invasive breast cancer, who completed both EQ-5D-5L and the Edmonton Symptom Assessment System. Participants were classified into five pre-defined health states, considered relevant both to clinical practice and economic modeling. We used the Canadian EQ-5D-5L value set to calculate community-valued health utility scores for each health state. Additionally, we evaluated aspects of construct validity (known-group and convergent validity) of EQ-5D-5L in breast cancer.

**Results:**

549 women were included; the mean age was 57 (SD 12) years. The mean EQ-5D-5L index score was 0.83 (SD 0.13; range 0.13 to 0.95), with a distribution skewed towards full health and a ceiling effect of 20%. The mean health utility value for early-stage breast cancer was 0.84 (95% CI 0.83–0.86) and for metastatic breast cancer was 0.78 (95% CI 0.76–0.81). This difference was 0.060 (lower 95% CI bound 0.036, slightly lower than the pre-specified minimum important difference of 0.037). Health utility values and ESAS scores met almost all pre-specified criteria for convergent validity.

**Conclusion:**

We generated a list of health utility values for five pre-defined breast cancer health states using EQ-5D-5L. Additional research is required to confirm the validity of EQ-5D-5L as an outcome measure in breast cancer.

**Supplementary Information:**

The online version contains supplementary material available at 10.1186/s41687-026-01044-x.

## Background

Breast cancer is the most frequent cancer among women globally [1] and the second most common cause of female cancer death in high-income countries [2, 3]. Breast cancer survival has improved significantly in the last decades as a result of screening programs and more effective treatments [4–7]. Currently, more than 80% of women with breast cancer have early-stage, highly curable disease at diagnosis [7]. 

Breast cancer is also a major focus of health technology assessment because it is common, often associated with long-term survivorship, and increasingly involves costly therapies delivered over extended periods [8]. Economic evaluations in breast cancer based on cost-utility analyses measure quality of life using health utility values. Health utility values are preference-based measures of health-related quality of life, typically reported on a 0 to 1 scale anchored at 1 (full health) and 0 (dead), and may take negative values for health states considered worse than dead [9, 10]. Societal preferences reflect how the general population value health states and can be used to compare health status across groups [11–14]. In economic evaluation, they are commonly combined with time spent in health states to estimate quality-adjusted life years (QALYs), an outcome measure commonly used in cost-utility analyses [15]. 

To inform these analyses, economic guidelines call for the use of generic preference-based health-related quality of life (HRQOL) instruments to measure societal preferences, with some guidelines endorsing the EuroQol 5-dimensions (EQ-5D) questionnaire, the most widely used generic preference-based HRQOL instrument [16–18]. The most recent version is the 5-level EQ-5D (EQ-5D-5L) [19]. 

Because the results of economic analyses are often sensitive to health utility values, high quality health utility data is essential for robust economic evaluation [20]. Small differences in health utility estimates can lead to meaningful differences in incremental QALYs and, consequently, cost-effectiveness analysis results [21]. Previous research has not yielded a single, generalizable set of health utility values for breast cancer [22, 23]. Our objective was to provide empirically derived and validated estimates of health utility values for pre-defined health states in a contemporary Canadian outpatient sample.

In research studies, health utility values vary significantly depending on the valuation method [24]. For example, standard gamble and time trade-off are direct preference elicitation methods that differ in their underlying assumptions about risk and time preferences [25]. Rating scales (e.g., visual analogue scales) are non-choice-based methods in which respondents rate health states on a bounded scale [26]. For early breast cancer, rating scales give the lowest health utility values and standard gamble the highest, but for metastatic breast cancer time trade-off gives the highest health utility values [22, 23]. These health utility values also vary by assessor (i.e., whose preferences are used to value a given health state description, whether vignette-based or instrument-defined), with patients giving higher values than clinicians or members of the general public for both early and metastatic breast cancer [22, 23, 27]. This is relevant because certain health technology assessment agencies require health utility values based on general population preferences rather than patient valuations [28–31]. Health utility values can also change over time, for example improving post-diagnosis for early-breast cancer without recurrence and declining substantially with disease progression for metastatic breast cancer [22]. Few studies have assessed health utility values for early breast cancer. For metastatic breast cancer, health utility values vary significantly by symptom severity, treatments, and side-effects [22]. 

Generic preference-based instruments, such as the EQ-5D, allow comparisons across diseases and interventions and are therefore well suited for economic evaluation, including in breast cancer [18, 32, 33]. They are also brief and straightforward to administer and score, which reduces respondent and administrative burden in clinical and research settings. However, they may be less sensitive to disease-specific concerns relevant to breast cancer, such as fatigue, body image, and sexual functioning, and may exhibit ceiling effects in relatively well-functioning outpatient populations [33, 34]. 

A key concept in quality-of-life assessment is construct validity – the assessment of whether an instrument is accurately representing what it is intended to measure. Construct validity can be assessed by determining if the instrument describes expected differences in scores between subgroups of patients (known-group or discriminative validity). Construct validity can also be assessed by testing hypotheses that are formulated based on existing knowledge about the relationships of scores on the instrument under study with scores on other instruments. The instruments may measure constructs that are similar (convergent validity) or dissimilar (divergent validity). Only a few studies have investigated the construct validity of EQ-5D in breast cancer patients, including studies from Korea and Iran (using an earlier version of the EQ-5D) and from Singapore (using the EQ-5D-5L) [35–38]. These studies generally reported acceptable validity and reliability of EQ-5D in breast cancer, with findings also suggesting that health utility estimates may differ across preference-based instruments and versions/languages. In this study, we focused on known-group and convergent validity as key aspects of construct validity that could be evaluated using our available measures.

The primary objectives of this study were: (1) to describe health-related quality of life in distinct breast cancer health states across the disease spectrum, using the EQ-5D-5L instrument; and (2) to analyze which patient and disease characteristics are associated with EQ-5D-5L scores. The secondary objective was to investigate the construct validity of the EQ-5D-5L in women with breast cancer.

## Methods

### Research design, setting and participants

We conducted a cross-sectional study, recruiting a convenience sample of women with invasive breast cancer during outpatient clinic visits to 2 cancer centres in Toronto, Canada. Participants were eligible for the study if they were: (1) over 17 years of age; (2) diagnosed with invasive breast cancer (stages I to IV); (3) undergoing treatment or active surveillance for breast cancer; and (4) English-literate. Participants were excluded if they could not give informed consent or complete the study instruments on their own. Each participant was asked to participate only once. Eligibility was restricted to participants with female sex recorded in clinical records (male breast cancer was excluded). Gender identity was not collected; throughout, we use the term “women” to refer to the sex category used for eligibility and to align with published population norms for women.

Participants were approached during their scheduled clinic visit. We attempted to recruit participants after they had completed a routinely administered symptom survey, the Edmonton Symptom Assessment System (ESAS) instrument, in waiting room kiosks. At one centre, the EQ-5D-5L was self-administered using electronic tablets (iPads^®^); at the other, it was self-administered in paper form. The EQ-5D-5L descriptive system and response options are identical across modes, and health utility values were derived using the same Canadian EQ-5D-5L value set for all participants. Measurement equivalence between paper and electronic EQ-5D-5L modes has been reported previously; however, administration mode was site-specific (not randomized) in this study [39]. Clinical and demographic data, as well as ESAS responses, were collected from participants’ charts. The Charlson Comorbidity Index was used to summarize comorbidity [40]. 

### Instruments

The EQ-5D consists of both a descriptive system and a visual analogue scale (EQ VAS) [41]. The descriptive system comprises 5 dimensions: mobility, self-care, usual activities, pain/discomfort, and anxiety/depression. Each dimension has 5 levels of perceived problems: no problems (level 1), slight problems (level 2), moderate problems (level 3), severe problems (level 4), and extreme problems (level 5). This version is termed the EQ-5D-5L; a previous version that used 3 levels is termed the EQ-5D-3L [19]. A unique health state is defined by one level from each of the five dimensions (for example, 11111, if no problems in all 5 dimensions). Each health state may be converted into a health utility value (a summary index) by applying a country-specific value set (tariff) that reflects general population preferences for each level in each dimension of the descriptive system. [42] We used Canadian EQ-5D-5L values reported by Xie et al. [42] The EQ VAS measures health-related quality of life using the respondents’ mark on a vertical, visual analogue scale numbered 0 to 100, where the endpoints are labelled “The best health you can imagine” (100) and “The worst health you can imagine” (0).

ESAS is a self-reported instrument, which measures nine common symptoms of cancer: pain, tiredness, drowsiness, nausea, lack of appetite, shortness of breath, depression, anxiety, and well-being [43, 44]. ESAS responses are scored on a scale from 0 (no symptoms) to 10 (worst possible symptom). ESAS scales include a physical distress score, an emotional distress score, and a total symptom distress score (SDS) [45–48]. The physical distress score, ranging from 0 to 60, incorporates pain, tiredness, nausea, drowsiness, lack of appetite and shortness of breath. The emotional distress score, ranging from 0 to 20, includes ESAS anxiety and depression. The total SDS incorporates the physical and emotional scores and the response to well-being and ranges from 0 to 90. Higher physical and total SDSs have been associated with shorter survival in a group of patients with advanced cancer, which included breast cancer patients [49]. 

### Classification into health states

We classified participants into five mutually exclusive health states, based on their disease status at the time of recruitment. Health states were pre-specified prior to analysis informed by a targeted review of published breast cancer health utility studies and health state structures commonly used in economic models, with the goal of defining pragmatic categories that reflect clinically meaningful phases of the disease trajectory (time since diagnosis / recurrence and metastatic status) and that are feasible to operationalize using routinely available clinical data [22, 50]. This approach was intended to support economic evaluation while also improving clinical comparability within each health state. The resulting health states were intended to be relevant both to clinical practice and economic modeling:

1. *“First year after primary breast cancer” (Health State 1)*: participants diagnosed with invasive breast cancer (stage I to III) in the 12 months prior to study enrolment and being treated with curative intent at the time of recruitment or before.
2. *“First year after recurrence or new primary breast cancer” (Health State 2)*: participants diagnosed with at least a second metachronous invasive breast cancer or a local-regional recurrence treated with curative intent, in the 12 months prior to study enrolment and without metastatic disease.
3. *“Second to fifth year after a primary breast cancer or recurrence treated with curative intent” (Health State 3)*: participants diagnosed with the latest primary invasive breast cancer or a loco-regional recurrence treated with curative intent more than one year, but fewer than five years, prior to study enrolment and without metastatic disease.
4. *“Sixth and following years after a primary breast cancer or recurrence treated with curative intent” (Health State 4)*: participants diagnosed with the latest primary invasive breast cancer or a loco-regional recurrence more than five years prior to study enrolment and without metastatic disease.
5. *“Metastatic Breast Cancer” (Health State 5)*: participants with distant or inoperable disease.

Classification was performed using chart-abstracted clinical information, including the date of the most recent primary diagnosis, the date of local-regional recurrence or new primary breast cancer, and the presence of metastatic disease at the time of recruitment. Metastatic disease status was assigned highest priority (i.e., participants with metastatic disease were classified as Health State 5 regardless of time since initial diagnosis). Among participants without metastatic disease, time since the most recent diagnosis / recurrence was used to define mutually exclusive phases, with the intent of reducing within-state heterogeneity using clinically interpretable criteria available in routine records. These health states were intended to represent phases of the breast cancer trajectory that are typically associated with different treatment intent and intensity, and therefore reflect treatments more commonly received within each phase (e.g., curative-intent surgery and/or radiotherapy, with or without systemic therapy, during the first year after primary breast cancer diagnosis). We characterized potential heterogeneity by reporting key demographic and clinical characteristics by health state and by examining associations between health utility values and patient- and disease-related factors (including comorbidity and treatment status at recruitment) in multivariable regression analyses.

### Hypotheses

For the primary objectives, we hypothesized that older patients, patients with greater comorbidity, patients with lower education, patients more recently diagnosed with breast cancer, patients with metastatic disease, and patients under treatment would have clinically meaningfully lower health utility values than patients without each characteristic. These hypotheses were informed by prior literature showing lower HRQOL and / or health utility values with more advanced or progressive disease, during or shortly after treatment, and among individuals with greater comorbidity and lower socioeconomic status. However, associations for some characteristics (e.g., age and time since diagnosis) have been mixed across studies [51–63]. We hypothesized lower health utility values with older age given the EQ-5D-5L’s emphasis on physical function and the higher burden of comorbidity with age.

We used an instrument-defined minimally important difference (MID) estimate for the Canadian EQ-5D-5L value set (0.037), derived from simulated single-level transitions. Although this estimate is not derived from a between-group comparison, we used it as a conservative benchmark for interpreting between-group differences in mean health utility values [64]. 

We evaluated construct validity through assessing known-group validity and convergent validity. For known-group validity, we tested the hypothesis that EQ-5D-5L could adequately discriminate between participants with metastatic disease (participants in health state 5, with the anticipated lowest mean health utility score) and early-stage disease (patients in health states 1, 2, 3 and 4, who were analyzed together). For these two groups, we evaluated whether the lower bound of the 95% confidence interval (95% CI) exceeded the MID of the Canadian scoring of the EQ-5D-5L [64]. 

We also explored whether HRQOL for early-stage disease was associated with time from initial diagnosis. To do so, we assessed whether: (1) the mean health utility value for health state 1 would be lower than for health state 3 and 4; and (2) the mean health utility value for health state 3 would be lower than for health state 4. We did not define a threshold for the differences between groups as these analyses were exploratory.

To assess convergent validity, we hypothesized that EQ-5D-5L mean health utility values for each health state would be at least moderately correlated with ESAS total SDS; we a priori defined moderate correlation as an absolute value of the Spearman correlation coefficient rho > 0.30 [65]. We also expected that: (1) the EQ-5D-5L dimensions of mobility, self-care, pain/discomfort and usual activities would be at least moderately correlated with ESAS physical score; (2) the EQ-5D-5L dimension of anxiety/ depression would be at least moderately correlated with ESAS emotional score; (3) the EQ-5D-5L dimension of pain/discomfort would be at least moderately correlated with ESAS pain; and (4) the EQ-5D-5L dimension of anxiety/ depression was expected to be at least moderately correlated with each of ESAS anxiety and ESAS depression. Construct validity was considered acceptable if the hypotheses of the primary analyses for known-group validity and convergent validity were both satisfied.

### Statistical analysis

We used descriptive statistics to characterize the study population. We reported the proportion of patients reporting each level in each EQ-5D-5L dimension by health state. Descriptive statistics (mean, 95% confidence interval, median, inter-quartile range [IQR], standard deviation [SD], skewness and kurtosis) were calculated for EQ-5D health utility values and VAS scores for each health state and for the whole patient population. Multivariable linear regression analysis was used to identify socio-demographic and clinical characteristics independently associated with EQ-5D-5L health utility values. Health state was included as a categorical (nominal) covariate to adjust for disease status. Ordinal regression was not used because EQ-5D-5L health utility values are continuous and the predefined health states are not inherently ordinal. To describe potential within-state heterogeneity, we also summarized key participant characteristics stratified by health state.

To test the hypotheses relating to known-group validity, we used the non-parametric Wilcoxon rank-sum (Mann-Whitney) test. To test the hypotheses for convergent validity, we performed Spearman’s correlation tests for each pair of measures.

Statistical analyses were conducted using SAS^®^ 9.4 (SAS Institute, Cary, NC, USA). All study procedures were approved by Sunnybrook and University Health Network Research Ethics Boards.

## Results

### Patient characteristics

A total of 549 participants who met the eligibility criteria were recruited (Table 1). The mean age of the participants was 57 (SD 12). Most participants had a Charlson Comorbidity Index of 0 (*N* = 396; 72%), were White (*N* = 321; 58%), born outside Canada (*N* = 300; 55%), and had an education level higher than high school (*N* = 442, 80.5%). Seventy-five percent (*N* = 412) had been diagnosed with breast cancer within 7 years of recruitment and were receiving active treatment for their cancer. ESAS data were available for 381 participants and were used for the construct validity analyses.

**Table 1:** Patient characteristics

Characteristics	*N* = 549 (%)
Age – Mean (SD; min-max)	57 (12; 25–90)
**Age**	
< 45 years	88 (16%)
45–64 years	313 (57%)
≥ 65 years	148 (27%)
**Menopausal Status**	
Pre-menopausal	146 (27%)
Post-menopausal	357 (65%)
Unsure/ Answer missing	46 (8%)
**Comorbidities (yes)**	420 (77%)
Anxiety and/or depression	122 (22%)
Arthritis	94 (17%)
Osteoporosis and/or history of fractures	86 (16%)
Hypertension	106 (19%)
Respiratory and/or cardiac disease	62 (11%)
Other cancers	45 (8%)
**Charlson Comorbidity Index**	
0	396 (72%)
1–2	132 (24%)
≥ 3	21 (4%)
**Born in Canada**	
Yes	238 (43%)
No	300 (55%)
Preferred not to answer	11 (2%)
**Annual Family Income** ^**a**^	
$0 to $59,999	142 (26%)
≥ $60,000	265 (48%)
Does not know/ Prefers not to answer	142 (26%)
**Education**	
Below Grade 8	15 (3%)
Attended / graduated high school	84 (15%)
Attended / graduated college/ university	313 (57%)
Postgraduate / professional	129 (24%)
Missing	8 (1%)
**Employment Status**	
Retired	166 (30%)
Unemployed	52 (9%)
Employed	242 (44%)
Other (e.g., on leave, disability)	86 (16%)
Missing	3 (1%)
**Marital Status**	
Married/ common law	370 (67%)
Separated/ divorced/ widowed	108 (20%)
Single/ Never married	65 (12%)
Missing	6 (1%)
**Primary Language spoken at home**	
English	393 (72%)
French	7 (1%)
Other	145 (26%)
**Racial or ethnic group**	
Asian – East / South East	109 (20%)
Asian – South	31 (6%)
Black / African Canadian	7 (1%)
Caribbean	22 (4%)
Latin American	11 (2%)
Middle Eastern	32 (6%)
Mixed heritage	3 (1%)
White	321 (58%)
Indigenous	2 (0.4%)
Preferred Not to Answer	11 (2%)
**Years living with invasive breast cancer - Median (IQR)**	2 (0.84–6.4)
**Year of Primary Diagnosis**	
1986–2000	38 (7%)
2001–2005	35 (6%)
2006–2010	64 (12%)
2011–2015	220 (40%)
2016–2017	192 (35%)
**Stage at diagnosis**	
Stage I	174 (32%)
Stage II	226 (41%)
Stage III	103 (19%)
Stage IV	40 (7%)
Unknown	6 (1%)
**Breast Cancer Subtype**	
Hormone-Receptor Positive	332 (60%)
HER2 Positive	109 (20%)
Triple negative	103 (19%)
Unknown	5 (1%)
**Previous Breast Surgery (yes)**	489 (89%)
**Current radiotherapy (yes)**	12 (2%)
**Current systemic therapy**	
Chemotherapy (+/- targeted therapy)	85 (15%)
Hormonal treatment (+/- targeted therapy)	291 (53%)
Targeted therapy (only)	24 (4%)
**Participating in a Clinical Trial (yes)**	18 (3%)
**Breast Cancer Health State**	
First year after primary breast cancer	146 (27%)
First year after recurrence or new primary breast cancer	13 (2%)
Second to fifth year after primary breast cancer or recurrence	185 (34%)
Sixth and following years after primary breast cancer or recurrence	62 (11%)
Metastatic Breast Cancer	143 (26%)

^a^ The study sites asked about income using different categories. These categories were collapsed and combined as shown. N= number; % = percentage; SD= standard deviation; IQR= interquartile range; CI= confidence interval; Min= minimum; Max= maximum

Participants were classified into 5 mutually exclusive breast cancer health states: (1) First year after primary breast cancer (*N* = 146; 27%); (2) First year after recurrence or new primary breast cancer (*N* = 13; 2%); (3) Second to fifth year after primary breast cancer or recurrence (*N* = 185; 34%); (4) Sixth and following years after primary breast cancer or recurrence (*N* = 62; 11%); Metastatic breast cancer (*N* = 143; 26%). Participant characteristics overall and by health state are presented in Table 1 and Additional File 1, respectively.

### Health-related quality of life by health state

**Fig. 1:**
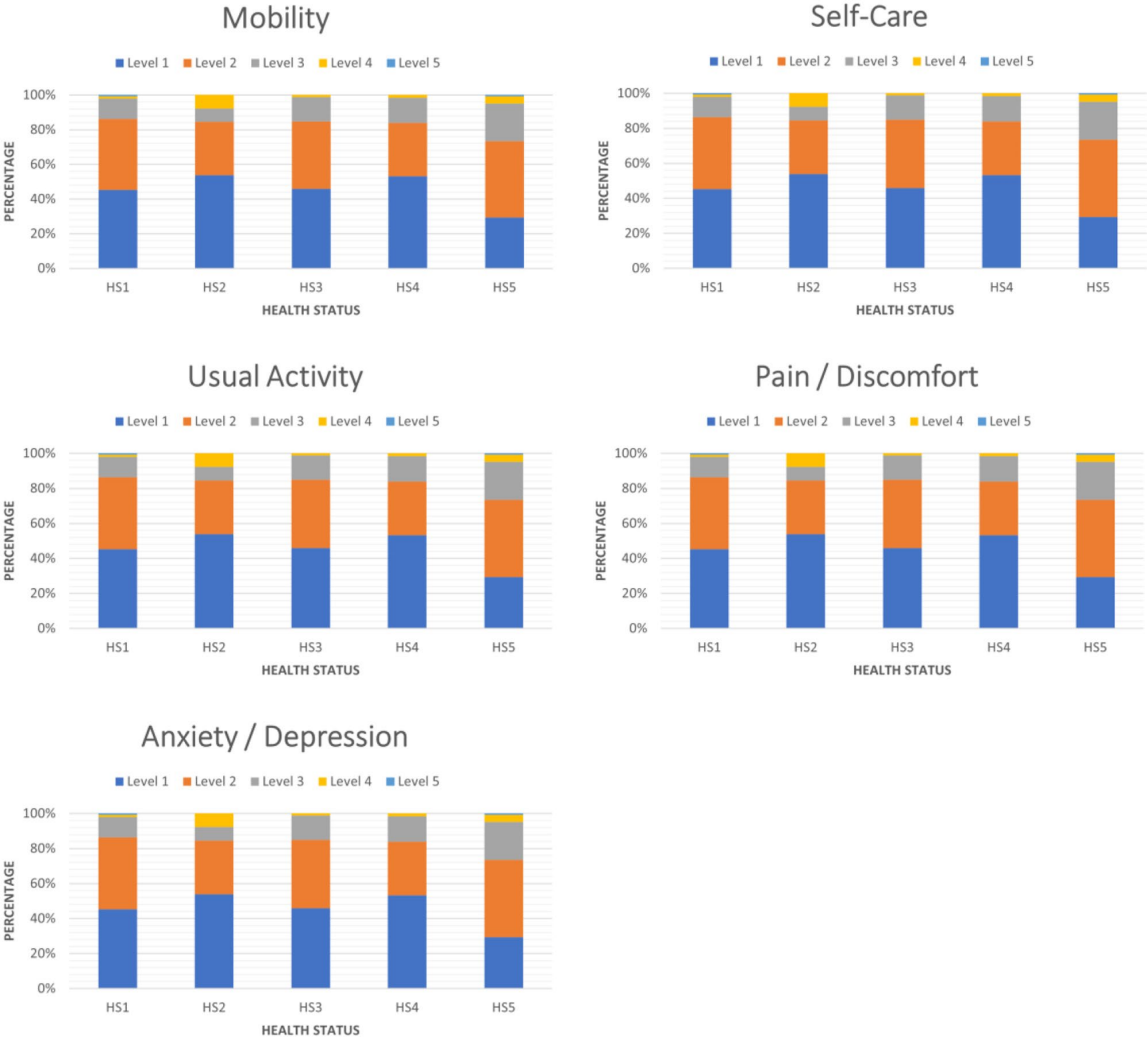
EQ-5D-5L Health profile by breast cancer health state. HS1= “First year after primary breast cancer”; HS2= “First year after recurrence or new primary breast cancer”; HS3=“Second to fifth year after a primary breast cancer or recurrence treated with curative intent”; HS4= “Sixth and following years after a primary breast cancer or recurrence treated with curative intent”; HS5= “Metastatic Breast Cancer”

Most participants reported “No problems” (scores of 1) in all the EQ-5D-5L dimensions, except for the “Pain/Discomfort” and “Anxiety/ Depression” dimensions, where more than 50% of the participants reported “Slight” to “Moderate” problems (Fig. 1 and Additional file 2). Very few participants reported “Severe” to “Extreme” problems in any dimension. Participants with metastatic breast cancer (Health State 5), reported the highest proportion of problems (scores greater than 1), particularly in the “Usual Activities”, “Pain/Discomfort” and “Anxiety/ Depression” dimensions.

We observed 126 distinct self-reported EQ-5D-5L health states in our patient population (of 3125 possible states), the best being “11111” = “no problems” in any of the EQ-5D-5L dimensions (110 participants, 20%) and the worst being “53322” = “extreme problems” on mobility, “moderate problems” on self-care and usual activities and “slight problems” on pain/discomfort and anxiety/depression (1 patient). (Additional file 3)

The mean EQ-5D-5L health utility value for all breast cancer participants was 0.83 (SD 0.13) (Table 2). Participants who had breast cancer treated with curative intent more than 5 years prior to enrollment (Health State 4) had the highest health utility value (mean 0.86; SD 0.14) and participants with metastatic breast cancer (Health State 5) had the lowest (mean 0.78; SD 0.14). Mean health utility values for Health States 1, 3, and 4 were similar, with overlapping confidence intervals (Table 2). Estimates for Health State 2 should be interpreted with caution given the small sample size (*N* = 13) and wide confidence interval. The mean EQ-5D-5L VAS value for all breast cancer patients was 75.0 (SD 17.5), with Health State 4 having highest values (mean 81.0; SD 15.6) and Health State 5 having the lowest (67.3; SD 19.2) (Additional file 4). The distribution of the health utility and VAS values was skewed towards full health (Additional files 5 and 6).

**Table 2:** EQ-5D-5L health utility values by breast cancer health state

Health State	*N* (%)	Mean (95%CI)	SD	Median (IQR)	Min-Max	Skewness	Kurtosis
HS 1	146 (27)	0.85 (0.83–0.86)	0.12	0.87 (0.83–0.91)	0.23–0.95	-2.81	10.26
HS 2	13 (2)	0.78 (0.65–0.90)	0.21	0.87 (0.76–0.89)	0.13–0.95	-2.75	8.63
HS 3	185 (34)	0.84 (0.83–0.86)	0.11	0.87 (0.81–0.91)	0.21–0.95	-2.08	6.53
HS 4	62 (11)	0.86 (0.82–0.89)	0.14	0.90 (0.85–0.95)	0.36–0.95	-2.20	4.54
HS 5	143 (26)	0.78 (0.76–0.81)	0.14	0.81 (0.70–0.90)	0.29–0.95	-1.18	1.09
Total	549 (100)	0.83 (0.82–0.84)	0.13	0.87 (0.80–0.91)	0.13–0.95	-2.01	5.05

HS1= “First year after primary breast cancer”; HS2= “First year after recurrence or new primary breast cancer”; HS3=“Second to fifth year after a primary breast cancer or recurrence treated with curative intent”; HS4= “Sixth and following years after a primary breast cancer or recurrence treated with curative intent”; HS5= “Metastatic Breast Cancer”; N= number; % = percentage; SD= standard deviation; IQR= interquartile range; CI= confidence interval; Min= minimum; Max= maximum. Estimates for Health State 2 should be interpreted with caution due to the small sample size (*N* = 13)

In the multivariable linear regression model, comorbidities and health state were associated with EQ-5D-5L health utility scores. A Charlson Comorbidity Index of 3 or higher (β= -0.067; 95% CI -0.125 to -0.008, *p* = 0.025) was associated with significantly lower health utility values than a Charlson Comorbidity Index of 0; Health States 1 (β = 0.065; 95% CI 0.018–0.111, *p* = 0.006), Health State 3 (β = 0.057; 95% CI 0.024–0.089, *p* = 0.001) and 4 (β = 0.069, 95% CI 0.028 to 0.111, *p* = 0.001) were associated with significantly higher health utility values than Health State 5 (Table 3).

**Table 3:** Linear regression model for EQ-5D-5L utility values

Predictor	*N* = 549 (%)	Adjusted coefficientes (95% CI)	*p*-value
**Age**			
< 45 years	88 (16%)	-0.004 (-0.042, 0.034)	0.830
45–64 years	313 (57%)	-0.006 (-0.033, 0.021)	0.656
≥ 65 years	148 (27%)	Reference	***-***
***Charlson Comorbidity Index***			
0	396 (72%)	Reference	-
1–2	132 (24%)	-0.020 (-0.047, 0.007)	0.140
≥ 3	21 (4%)	-0.067 (-0.125, -0.008)	0.025
***Education***			
Grade 8 education or less	15 (3%)	-0.036 (-0.105, 0.034)	0.316
Some or completed High school	84 (16%)	-0.020 (-0.046, 0.007)	0.147
Some or completed college or university	313 (58%)	-0.024 (-0.060, 0.012)	0.198
Some or completed postgraduate / professional	129 (24%)	Reference	***-***
***Year of Primary Diagnosis***			
1986–2000	38 (9%)	0.004 (-0.056, 0.063)	0.905
2001–2005	35 (6%)	-0.007 (-0.068, 0.054)	0.819
2006–2010	64 (12%)	0.003 (-0.051, 0.056)	0.919
2011–2015	220 (40%)	0.004 (-0.035, 0.044)	0.827
2016–2017	192 (35%)	Reference	***-***
***Breast Cancer Subtype***			
Hormone-Receptor Positive	332 (61%)	0.006 (-0.023, 0.034)	0.687
Triple negative	103 (19%)	0.020 (-0.016, 0.056)	0.280
HER2 Positive	109 (20%)	Reference	***-***
***Breast Cancer Health State***			
HS 1	146 (27%)	0.065 (0.018, 0.111)	0.006
HS 2	13 (2%)	-0.001 (-0.075, 0.073)	0.980
HS 3	185 (34%)	0.057 (0.024, 0.089)	0.001
HS 4	62 (11%)	0.069 (0.028, 0.111)	0.001
HS 5	143 (26%)	Reference	***-***
***Current radiotherapy***			
Yes	12 (2%)	Reference	-
No	537 (98%)	-0.004 (-0.079, 0.071)	0.913
***Current systemic therapy***			
Yes	400 (73%)	Reference	-
No	149 (27%)	-0.016 (-0.042, 0.010)	0.225

HS1= “First year after primary breast cancer”; HS2= “First year after recurrence or new primary breast cancer”; HS3=“Second to fifth year after a primary breast cancer or recurrence treated with curative intent”; HS4= “Sixth and following years after a primary breast cancer or recurrence treated with curative intent”; HS5= “Metastatic Breast Cancer”; N= number; % = percentage

### Known-group (discriminative) validity

The mean health utility value for early-stage breast cancer (Health States 1, 2, 3 and 4) was 0.84 (95% CI 0.83–0.86), compared with 0.78 (95% CI 0.76–0.81) for metastatic breast cancer (Health State 5). The difference in mean health utility values was 0.060 (95% CI 0.036 to 0.085), with the lower bound of the confidence interval approximating the pre-specified MID (0.037).

In exploratory analyses, mean health utility values were similar across early-stage health states. The mean health utility value for participants in the first year after primary breast cancer diagnosis (Health State 1), and participants in their second to fifth year after a primary breast cancer treated with curative intent (Health State 3) were similar (difference of means 0.001, 95% CI -0.024 to 0.026). The mean health utility value for participants in Health State 1 and participants in their sixth and following years after a primary breast cancer treated with curative intent (Health State 4) were also similar (difference of means − 0.012, 95% CI -0.049 to 0.026). EQ-5D-5L was similar for participants in Health State 3 and Health State 4 (difference of means − 0.013, 95% CI -0.047 to 0.022). Estimates for Health State 2 should be interpreted with caution given the small sample size.

### Convergent validity

We observed a negative correlation between health utility values and ESAS physical, emotional, and total SDS scores, indicating lower health utility values with greater symptom burden (Table 4). Absolute values of EQ-5D-5L and ESAS total SDSs correlation coefficients were significantly higher than 0.30 for all health states (Table 4). There was a positive correlation between EQ-5D-5L dimension scores and corresponding ESAS summary and symptom score (higher EQ-5D-5L dimension scores indicate worse health) (Table 5). Correlation coefficients were significantly higher than 0.30 for all of those comparisons, except for EQ-5D-5L self-care / ESAS physical score. There was also a positive correlation between EQ-5D-5L dimension scores for anxiety/depression and ESAS emotional score, ESAS Anxiety and ESAS Depression. (Table 5), with correlation coefficients significantly higher than 0.30.

**Table 4:** Spearman correlation coefficients between EQ-5D-5L health utility values and ESAS (Edmonton Symptom Assessment System) physical score, emotional score and total symptom distress score (SDS)

Health State	*N* (%)	ESAS Physical Score (95% CI)	ESAS Emotional Score (95% CI)	ESAS Total SDS (95% CI)
Health State 1	99 (26%)	-0.55 (-0.68, -0.40)	-0.50 (-0.64, -0.34)	-0.61 (-0.72, -0.47)
Health State 2	8 (2%)	-0.87 (-0.98, -0.44)	-0.21 (-0.79, 0.58)	-0.76 (-0.95, -0.12)
Health State 3	124 (33%)	-0.59 (-0.70, -0.47)	-0.49 (-0.62, -0.35)	-0.64 (-0.73, -0.52)
Health State 4	47 (12%)	-0.79 (-0.88, -0.65)	-0.75 (-0.86, -0.60)	-0.82 (-0.90, -0.70)
Health State 5	103 (27%)	-0.65 (-0.75, -0.52)	-0.50 (-0.63, -0.34)	-0.68 (-0.78, -0.57)
Total	381	-0.65 (-0.71, -0.59)	-0.53 (-0.60, -0.45)	-0.69 (-0.74, -0.63)

Health State 1= “First year after primary breast cancer”; Health State 2= “First year after recurrence or new primary breast cancer”; Health State 3=“Second to fifth year after a primary breast cancer or recurrence treated with curative intent”; Health State 4= “Sixth and following years after a primary breast cancer or recurrence treated with curative intent”; Health State 5= “Metastatic Breast Cancer”; N= number; % = percentage; CI= confidence interval

**Table 5:** Spearman correlation coefficients between EQ-5D-5L dimensions and selected ESAS (Edmonton Symptom Assessment System) summary and symptom scores

EQ-5D-5L Dimensions (*N* = 549)	ESAS Physical Score (*N* = 381) (95% CI)	ESAS Emotional Score (*N* = 381) (95% CI)	ESAS Pain (*N* = 380)	ESAS Anxiety (*N* = 380)	ESAS Depression (*N* = 380)
Mobility	0.45 (0.36, 0.53)	-	-	-	-
Self-care	0.31 (0.22, 0.40)	-	-	-	-
Usual activities	0.52 (0.44, 0.59)	-	-	-	-
Pain/ discomfort	0.59 (0.52, 0.65)	-	0.73 (0.68, 0.78)	-	-
Anxiety / depression	-	0.78 (0.74, 0.82)	-	0.71 (0.66, 0.76)	0.70 (0.64, 0.74)

N= number; % = percentage; CI= confidence interval. Correlations were calculated among participants with both EQ-5D-5L and ESAS data available, depending on missingness

## Discussion and conclusions

We used the EQ-5D-5L to determine health utility values for five distinct breast cancer health states, which span the disease spectrum and are relevant to both clinical practice and economic modeling. Our study is one of the first to apply the latest version of the EQ-5D to study participants with breast cancer and to include a contemporary population receiving current treatment protocols.

Overall, we found a mean EQ-5D-5L value of 0.83 (95%CI 0.82 to 0.84), with values of 0.86 (95%CI 0.82 to 0.89) for participants in the most favorable health state (sixth and following years after a primary breast cancer or recurrence treated with curative intent) and 0.79 (95%CI 0.76 to 0.81) for participants in the least favorable health state (metastatic breast cancer). For comparison, the population norm for Canadian women has been estimated at 0.869, suggesting that women in our sample without metastatic disease have a favorable quality of life profile after treatment [66]. On the other hand, the population norm for Canadian women with any self-reported chronic health condition is 0.84 (mean VAS 79.3), with our results suggesting that women with breast cancer in our sample have worse quality of life during treatment and with metastatic disease [66]. A previous Swedish study which also included breast cancer patients in different disease states, reported lower EQ-5D-3L mean health utility values for the overall population (0.695, 95% CI 0.634 to 0.747) and for specific disease states, using the UK value set [50]. A possible explanation for the observed differences, apart from differences in patient characteristics and value set used, is that breast cancer treatments have evolved in the years between that study and ours.

Most participants in our study did not report any problems in any of the five EQ-5D-5L dimensions that make up the EQ-5D profiles. Such ceiling effects exist when scores cluster at the high end of a scale. While strong ceiling effects may limit the validity of an instrument, our observed effect (20%) was lower than that reported for a Canadian population (33.1%) using EQ-5D-5L [66]. A study in general population suggested that EQ-5D-5L ceiling effects are negatively correlated with morbidity, with the probability of indicating health state “11111” falling below the 15% if ≥ 3 diseases were present [67]. High ceiling effects might be a result of people being “healthy” in the dimensions evaluated by the EQ-5D-5L. Therefore, disease-specific HRQOL instruments should be used together with preference-based instruments when assessing individual health status [67, 68]. In breast cancer, generic instruments such as EQ-5D-5L may not fully capture domains that are commonly affected, including fatigue, cognitive symptoms, sexual functioning, and body image, which may reduce sensitivity to differences across clinical subgroups [34, 69]. 

The symptom scores for “Pain/Discomfort” and “Anxiety/ Depression” were the most frequently rated as problematic, including by participants who were several years after initial diagnosis (Health States 3 and 4). Those EQ-5D domains have previously been reported as the main drivers behind the reduction in HRQOL in breast cancer patients [50]. In contrast, dimensions such as mobility and self-care showed relatively few reported problems in this outpatient sample, which may contribute to ceiling effects and limit discrimination among early-stage health states. This likely reflects preserved physical functioning in this outpatient clinic population. In the multivariable analysis, we found that EQ-5D-5L health utility values were associated with health state and the number of comorbidities, which underscores the importance of considering those as factors associated with health-related quality of life. Year of diagnosis, breast cancer subtype, and receipt of radiotherapy or systemic therapy at the time of recruitment, were not strongly associated with EQ-5D-5L health utility values after adjusting for breast cancer health state and comorbidities. These findings differ from older studies that demonstrated lower health-related quality of life in breast cancer participants in the first year after diagnosis, particularly for younger participants (≤ 50 years old), participants who were receiving treatment (chemotherapy, radiation or tamoxifen), participants with shorter duration of disease since diagnosis, participants with lower education and socio-economic groups, and older participants (over-time) [51–60, 70]. Our findings are more consistent with long-term studies that show that disease-free survivors report levels of physical, emotional, and social functioning comparable to the general population, and that HRQOL is lower with more advanced stage at diagnosis, progressive disease and higher number of comorbidities [61, 62, 71–73]. 

We found good evidence for convergent validity, with 6 of 7 tests meeting our pre-specified criteria. However, we found limited evidence for known group/ discriminative validity. The confidence interval for the difference in mean health utility value between early stage and metastatic breast cancer states (0.036 to 0.085) included the pre-specified minimum important difference of 0.037, suggesting that EQ-5D-5L may not effectively discriminate between those breast cancer states in this outpatient sample. Another possible explanation is that the analysis might have been underpowered due to a potentially inadequate sample size, limiting our ability to detect differences between early stage and metastatic breast cancer states. We also found no differences among mean health utility values for the defined early-stage breast cancer health states. Although it is possible that no differences exist between them, it is unlikely that no difference in quality of life among these participants exists, given the clinic context. Therefore, the results underscore the need for further research to clarify these findings. Prior studies using other instruments (FACT-B and SF-6D) to investigate convergent validity with the EQ-5D did not report or test a priori hypotheses to assess construct validity [35–37]. Future studies should pre-specify hypotheses, include a larger and more diverse patient population, and investigate potential confounding factors. Given the modest differences in mean health utility values across predefined health states and the potential heterogeneity within states, our findings should be interpreted primarily as descriptive estimates for these pragmatic categories. Future studies may benefit from defining more homogeneous health states and/or focusing on modeling health utility values using explanatory factors rather than discrete health state categories. Future work should also compare the discriminative ability and responsiveness of EQ-5D-5L with cancer-specific preference-based measures (e.g., EORTC QLU-C10D), which include cancer-relevant domains (e.g., fatigue) that are not explicitly captured in the EQ-5D-5L [74]. Comparative studies in breast cancer are warranted to assess discrimination and responsiveness over time [75]. 

Our study has limitations. In retrospect, an alternative health state definition may have provided better results, given the small sample size of health state 2 and the observed similarity of mean health utility values across health states. For example, an alternative a priori approach could collapse early-stage categories into fewer, better powered states aligned with common economic models (e.g., early-stage on/within 12 months of active treatment versus early-stage post-treatment survivorship) or combine Health State 2 with other curative intent health states. We only recruited participants in two academic centres in Toronto, who might not be representative of the population of individuals with breast cancer. We did not recruit participants who were too unwell to be seen in an outpatient clinic (for example, patients admitted to hospital or to palliative care). We also did not recruit long-term survivors, who might still be dealing with side effects of cancer treatments. 58% of the participants were White and more than 80% had an education level higher than high school, which might also limit the generalizability of our findings. In addition, eligibility required English literacy, which may have excluded individuals with limited English proficiency and may reduce generalizability to populations in whom language barriers are associated with different symptom reporting and access to supportive care, and related health utility values [76]. Finally, EQ-5D-5L was administered electronically at one site and on paper at the other; administration mode was not randomized and may contribute to measurement variability. We were not able to consider specific cancer treatments when reporting health utility values, given the complexities regarding different treatments each participant could be receiving in each health state. In addition, heterogeneity in treatment pathways and symptom burden within each health state may limit interpretation of regression coefficients; therefore, regression findings should be interpreted as exploratory associations rather than causal effects. However, treatment effects were implicitly captured through symptom scores [23, 50, 77]. 

In conclusion, we generated a list of health utility values for five pre-defined breast cancer health states using EQ-5D-5L. While some of our results provide supportive evidence for construct validity (particularly convergent validity) of EQ-5D-5L in breast cancer, additional research is required to confirm discriminative validity across clinically relevant subgroups and to assess responsiveness over time. Our results will also help researchers to decide how to define breast-cancer- related health states for future health utility studies, while recognizing that differences between early-stage health states were modest in this outpatient sample.

## Supplementary Information

Below is the link to the electronic supplementary material.

Additional File 1.pdf- Patient Characteristics by health state. The study sites asked about income using different categories. These categories were collapsed and combined as shown. HS1= “First year after primary breast cancer”; HS2= “First year after recurrence or new primary breast cancer”; HS3=“Second to fifth year after a primary breast cancer or recurrence treated with curative intent”; HS4= “Sixth and following years after a primary breast cancer or recurrence treated with curative intent”; HS5= “Metastatic Breast Cancer”, N= number; % = percentage; SD= standard deviation; IQR= interquartile range; CI= confidence interval; Min= minimum; Max= maximum

Additional File 2.pdf- Table of EQ-5D-5L Health Profile results by breast cancer health state. HS1= “First year after primary breast cancer”; HS2= “First year after recurrence or new primary breast cancer”; HS3=“Second to fifth year after a primary breast cancer or recurrence treated with curative intent”; HS4= “Sixth and following years after a primary breast cancer or recurrence treated with curative intent”; HS5= “Metastatic Breast Cancer”.; Level 1= “No problems”, Level 2= “Slight problems”, Level 3= “Moderate problems”; Level 4= “Severe problems”; Level 5= “Extreme problems”

Additional File 3.pdf- Table with the prevalence of the 6 most frequently observed self-reported health states and frequency of reporting of the best and worst health state profile in each health state. HS1= “First year after primary breast cancer”; HS2= “First year after recurrence or new primary breast cancer”; HS3=“Second to fifth year after a primary breast cancer or recurrence treated with curative intent”; HS4= “Sixth and following years after a primary breast cancer or recurrence treated with curative intent”; HS5= “Metastatic Breast Cancer”

Additional File 4.pdf- Table with EQ-5D-5L VAS values by breast cancer health state. HS1= “First year after primary breast cancer”; HS2= “First year after recurrence or new primary breast cancer”; HS3=“Second to fifth year after a primary breast cancer or recurrence treated with curative intent”; HS4= “Sixth and following years after a primary breast cancer or recurrence treated with curative intent”; HS5= “Metastatic Breast Cancer”; Std Dev= standard deviation; IQR= interquartile range; CI= confidence interval. Six patients did not complete this part of the instruments

Additional File 5.tif- Histogram for EQ-5D-5L health utility values

Additional File 6.tif- Histogram for EQ-5D-5L VAS values

## Data Availability

All data generated or analysed during this study are included in this published article and its supplementary information files.
