# Supplementary material for: Health-related quality of life in breast cancer measured with EQ-5D-5L
Source: J Patient Rep Outcomes. 2026 Mar 20;10:67. doi: 10.1186/s41687-026-01044-x (PMC13125455; doi:10.1186/s41687-026-01044-x)
Supplement: Supplementary file 1 — Additional File 1.pdf- Patient Characteristics by health state. The study sites asked about income using different categories. These categories were collapsed and combined as shown. HS1= “First year after primary breast cancer”; HS2= “First year after recurrence or new primary breast cancer”; HS3=“Second to fifth year after a primary breast cancer or recurrence treated with curative intent”; HS4= “Sixth and following years after a primary breast cancer or recurrence treated with curative intent”; HS5= “Metastatic Breast Cancer”, N= number; % = percentage; SD= standard deviation; IQR= interquartile range; CI= confidence interval; Min= minimum; Max= maximum [file 41687_2026_1044_MOESM1_ESM.pdf]

Characteristics	HS1 (N=146)	HS2 (N=13)	HS3 (N=185)	HS4 (N=62)	HS5 (N=143)
Age – Mean (SD; min-max)	55 (13; 25-90)	65 (10; 43-78)	56 (13; 28-87)	60 (9; 38-81)	58 (11; 32-81)
**Menopausal Status**					
Pre-menopausal	54 (37%)	1 (8%)	62 (34%)	6 (10%)	23 (16%)
Post-menopausal	77 (53%)	12 (92%)	112 (61%)	48 (77%)	108 (76%)
Unsure/ Answer missing	15 (10%)	0 (0%)	11 (6%)	8 (13%)	12 (8%)
**Charlson Comorbidity Index**					
0	118 (81%)	8 (62%)	134 (72%)	48 (77%)	88 (62%)
1-2	23 (16%)	4 (31%)	45 (24%)	12 (19%)	48 (34%)
≥3	5 (3%)	1 (8%)	6 (3%)	2 (3%)	6 (4%)
**Born in Canada**					
Yes	54 (37%)	5 (38%)	89 (48%)	25 (40%)	65 (45%)
No	88 (60%)	8 (62%)	93 (50%)	34 (55%)	77 (54%)
Preferred not to answer	4 (3%)	0 (0%)	3 (2%)	3 (5%)	1 (1%)
**Annual Family Income**					
$0 to $59,999	39 (27%)	3 (23%)	39 (21%)	20 (32%)	41 (29%)
≥ $60,000	71 (49%)	9 (69%)	99 (54%)	24 (39%)	62 (43%)
Does not know/ Prefers not to answer	36 (25%)	1 (8%)	47 (25%)	18 (29%)	40 (28%)
**Education**					
Below Grade 8	6 (4%)	0 (0%)	5 (3%)	1 (2%)	3 (2%)
Attended / graduated high school	22 (15%)	11 (85%)	22 (12%)	28 (45%)	24 (17%)
Attended / graduated college/ university	77 (53%)	2 (15%)	108 (58%)	14 (23%)	89 (62%)
Postgraduate / professional	38 (26%)	0 (0%)	47 (25%)	19 (31%)	25 (17%)
Missing	3 (2%)	0 (0%)	3 (2%)	0 (0%)	2 (1%)
**Employment Status**					
Retired	40 (27%)	4 (31%)	48 (26%)	24 (39%)	50 (35%)
Unemployed	15 (10%)	2 (15%)	14 (8%)	1 (2%)	20 (14%)
Employed	65 (45%)	6 (46%)	97 (52%)	33 (53%)	41 (29%)
Other (e.g, on leave, disability)	25 (17%)	1 (7%)	26 (14%)	3 (5%)	31 (22%)
Missing	1 (1%)	0 (0%)	0 (0%)	1 (2%)	1 (1%)

**Marital Status**					
Married/ common law	99 (68%)	9 (69%)	125 (68%)	43 (69%)	94 (66%)
Separated/ divorced/ widowed	28 (19%)	3 (23%)	35 (19%)	13 (21%)	29 (20%)
Single/ Never married	16 (11%)	1 (8%)	25 (14%)	5 (8%)	18 (13%)
Missing	3 (2%)	0 (0%)	0 (0%)	1 (2%)	2 (1%)
**Primary Language spoken at home**					
English	105 (72%)	12 (92%)	136 (74%)	38 (61%)	102 (71%)
French	2 (1%)	0 (0%)	2 (1%)	1 (2%)	2 (1%)
Other	37 (25%)	1 (8%)	46 (25%)	22 (35%)	39 (27%)
**Racial or ethnic group**					
Asian – East / South East	37 (25%)	2 (15%)	34 (18%)	10 (16%)	26 (18%)
Asian – South	9 (6%)	1 (8%)	8 (4%)	3 (5%)	10 (7%)
Black / African Canadian	2 (1%)	0 (0%)	2 (1%)	0 (0%)	3 (2%)
Caribbean	6 (4%)	0 (0%)	9 (5%)	2 (3%)	5 (4%)
Latin American	2 (1%)	0 (0%)	5 (3%)	1 (2%)	3 (2%)
Middle Eastern	6 (4%)	2 (15%)	11 (6%)	8 (13%)	5 (4%)
Mixed heritage	2 (1%)	0 (0%)	0 (0%)	0 (0%)	1 (1%)
White	77 (53%)	8 (62%)	114 (62%)	37 (60%)	85 (60%)
Indigenous	0 (0%)	0 (0%)	1 (1%)	0 (0%)	1 (1%)
Preferred Not to Answer	5 (3%)	0 (0%)	1 (2%)	1 (2%)	4 (3%)
**Years living with invasive breast cancer - Median (IQR)**	0.47 (0.30)	15 (10)	3 (3)	9 (5)	9 (7)
**Year of Primary Diagnosis**					
1986-2000	0 (0)	4 (31%)	4 (2%)	6 (10%)	24 (17%)
2001-2005	0 (0%	4 (31%)	3 (2%)	8 (13%)	20 (14%)
2006-2010	0 (0%)	2 (15%)	1 (1%)	31 (50%)	30 (21%)
2011-2015	2 (1%)	2 (15%)	147 (79%)	17 (27%)	52 (36%)
2016-2017	144 (99%)	1 (8%)	30 (16%)	0 (0%)	17 (12%)
**Stage at diagnosis**					
Stage I	54 (37%)	10 (77%)	69 (37%)	24 (39%)	17 (12%)
Stage II	71 (49%)	2 (15%)	86 (46%)	24 (39%)	43 (30%)

Stage III	21 (14%)	1 (8%)	29 (16%)	14 (23%)	39 (27%)
Stage IV	0 (0%)	0 (0%)	0 (0%)	0 (0%)	39 (27%)
Unknown	0 (0%)	0 (0%)	1 (0%)	0 (0%)	5 (3%)
**Breast Cancer Subtype**					
Hormone-Receptor Positive	85 (58%)	7 (54%)	123 (66%)	30 (48%)	87 (61%)
HER2 Positive	33 (23%)	3 (23%)	40 (22%)	20 (32%)	32 (22%)
Triple negative	27 (18%)	3 (23%)	21 (11%)	11 (18%)	22 (15%)
Unknown	1 (1%)	0 (0%)	1 (2%)	1 (2%)	2 (1%)
**Previous Breast Surgery (yes)**	120 (82%)	13 (100%)	185 (100%)	62 (100%)	109 (76%)
**Current radiotherapy (yes)**	8 (5%)	1 (8%)	0 (0%)	0 (0%)	3 (2%)
**Current systemic therapy**					
Chemotherapy (+/- targeted therapy)	45 (31%)	4 (31%)	0 (0%)	0 (0%)	36 (25%)
Hormonal treatment (+/- targeted therapy)	39 (27%)	6 (46%)	31 (50%)	31 (50%)	79 (55%)
Targeted therapy (only)	8 (5%)	0 (0%)	6 (3%)	2 (3%)	8 (6%)
**Participating in a Clinical Trial (yes)**	2 (1%)	0 (0%)	1 (1%)	1 (1%)	14 (10%)
