# Supplementary material for: Health-related quality of life in breast cancer measured with EQ-5D-5L
Source: J Patient Rep Outcomes. 2026 Mar 20;10:67. doi: 10.1186/s41687-026-01044-x (PMC13125455; doi:10.1186/s41687-026-01044-x)
Supplement: Supplementary file 2 — Additional File 2.pdf- Table of EQ-5D-5L Health Profile results by breast cancer health state. HS1= “First year after primary breast cancer”; HS2= “First year after recurrence or new primary breast cancer”; HS3=“Second to fifth year after a primary breast cancer or recurrence treated with curative intent”; HS4= “Sixth and following years after a primary breast cancer or recurrence treated with curative intent”; HS5= “Metastatic Breast Cancer”.; Level 1= “No problems”, Level 2= “Slight problems”, Level 3= “Moderate problems”; Level 4= “Severe problems”; Level 5= “Extreme problems” [file 41687_2026_1044_MOESM2_ESM.pdf]

EQ-5D Dimensions		Health States					Total
		**HS1** (N=146)	**HS2** (N=13)	**HS3** (N=185)	**HS4** (N=62)	**HS5** (N=143)	(N=549)
**Mobility**	*Level 1*	111; 76%	9; 69%	132; 71%	46; 74%	74; 52%	372; 68%
	Level 2	26; 18%	3; 23%	38; 21%	10; 16%	44; 31%	121; 22%
	Level 3	8; 5%	1; 8%	13; 7%	3; 5%	20; 14%	45; 8%
	Level 4	1; 1%	-	2;1%	2; 3%	4; 3%	9; 2%
	Level 5	-	-	-	1; 2%	1; 1%	2; 0.4%
**Self-Care**	*Level 1*	124; 85%	10; 77%	167; 90%	57; 92%	114; 80%	472; 86%
	Level 2	18; 12%	2; 15%	15; 8%	3; 5%	23; 16%	61; 11%
	Level 3	3; 2%	1; 8%	3; 2%	2; 3%	5; 4%	14; 3%
	Level 4	-	-	-	-	-	-
	Level 5	1; 1%	-	-	-	1; 1%	2; 0.4%
**Usual Activity**	*Level 1*	67; 46%	6; 46%	112; 61%	48; 77%	55; 38%	288; 52%
	Level 2	55; 38%	3; 23%	53; 29%	7; 11%	55; 38%	173; 32%
	Level 3	21; 14%	3; 23%	19; 10%	7; 11%	29; 20%	79; 14%
	Level 4	2; 1%	1; 8%	1; 1%	-	2; 1%	6; 1%
	Level 5	1; 1%	-	-	-	2; 1%	3; 1%
**Pain/ Discomfort**	*Level 1*	63; 43%	1; 8%	61; 33%	34; 55%	38; 27%	197; 36%
	Level 2	63; 43%	8; 62%	90; 49%	16; 26%	57; 40%	234; 43%
	Level 3	17; 12%	2; 15%	26; 14%	10; 16%	40; 28%	95; 17%
	Level 4	2; 1%	2; 15%	8; 4%	1; 2%	6; 4%	19; 4%
	Level 5	1; 1%	-	-	1; 2%	2; 1%	4; 1%
**Anxiety/ Depression**	*Level 1*	66; 45%	7; 54%	85; 46%	33; 53%	42; 29%	233; 42%
	Level 2	60; 41%	4; 31%	72; 39%	19; 31%	63; 44%	218; 40%
	Level 3	17; 12%	1; 8%	26; 14%	9; 15%	31; 22%	84; 15%
	Level 4	2; 1%	1; 8%	2; 1%	1; 2%	6; 4%	12; 2%
	Level 5	1; 1%	-	-	-	1;1%	2; 0.4%
