# Supplementary material for: Health-related quality of life in breast cancer measured with EQ-5D-5L
Source: J Patient Rep Outcomes. 2026 Mar 20;10:67. doi: 10.1186/s41687-026-01044-x (PMC13125455; doi:10.1186/s41687-026-01044-x)
Supplement: Supplementary file 3 — Additional File 3.pdf- Table with the prevalence of the 6 most frequently observed self-reported health states and frequency of reporting of the best and worst health state profile in each health state. HS1= “First year after primary breast cancer”; HS2= “First year after recurrence or new primary breast cancer”; HS3=“Second to fifth year after a primary breast cancer or recurrence treated with curative intent”; HS4= “Sixth and following years after a primary breast cancer or recurrence treated with curative intent”; HS5= “Metastatic Breast Cancer” [file 41687_2026_1044_MOESM3_ESM.pdf]

<table>
<tr><td colspan="3">EQ-5D-5L most observed profiles</td><td colspan="2">Frequency (%)</td></tr>
<tr><td colspan="3">11111</td><td colspan="2">110 (20%)</td></tr>
<tr><td colspan="3">11122</td><td colspan="2">44 (8%)</td></tr>
<tr><td colspan="3">11112</td><td colspan="2">40 (7%)</td></tr>
<tr><td colspan="3">11121</td><td colspan="2">40 (7%)</td></tr>
<tr><td colspan="3">11222</td><td colspan="2">36 (7%)</td></tr>
<tr><td colspan="3">21222</td><td colspan="2">23 (4%)</td></tr>
<tr><td>Health State 1</td><td>Health State 2</td><td>Health State 3</td><td>Health State 4</td><td>Health State 5</td></tr>
<tr><td>Best: 11111
(n=26; 17.8%)
Worst: 42442
(n=1; 0.7%)</td><td>Best: 11111
(n=1; 7,7%)
Worst: 33444
(n=1, 7,7%)</td><td>Best: 11111
(n=44; 23,8%)
Worst: 43443
(n=1; 0,5%)</td><td>Best: 11111
(n=23; 37,1%)
Worst: 51153
(n=1; 1,6%)</td><td>Best: 11111
(n=16; 11,1%)
Worst: 53322
(n=1; 0,7%)</td></tr>
</table>
