# Supplementary material for: Health-related quality of life in breast cancer measured with EQ-5D-5L
Source: J Patient Rep Outcomes. 2026 Mar 20;10:67. doi: 10.1186/s41687-026-01044-x (PMC13125455; doi:10.1186/s41687-026-01044-x)
Supplement: Supplementary file 4 — Additional File 4.pdf- Table with EQ-5D-5L VAS values by breast cancer health state. HS1= “First year after primary breast cancer”; HS2= “First year after recurrence or new primary breast cancer”; HS3=“Second to fifth year after a primary breast cancer or recurrence treated with curative intent”; HS4= “Sixth and following years after a primary breast cancer or recurrence treated with curative intent”; HS5= “Metastatic Breast Cancer”; Std Dev= standard deviation; IQR= interquartile range; CI= confidence interval. Six patients did not complete this part of the instruments [file 41687_2026_1044_MOESM4_ESM.pdf]

Health State	N (%)	Mean (95% CI)	Std Dev	Median (IQR)	Min-Max	Skewness	Kurtosis
**HS 1**	145 (27)	76.5 (73.8-79.3)	16.6	80.0 (68.0-90.0)	10-100	-1.06	1.52
**HS 2**	12 (2)	68.9 (54.1-83.7)	23.3	76.5 (52.5-84.5)	20-100	-0.85	0.19
**HS 3**	182 (34)	78.3 (76.1-80.4)	14.7	80.0 (70.0-90.0)	36-100	-0.69	-0.03
**HS 4**	61 (11)	81.0 (77.1-85.0)	15.6	82.0 (75.0-92.0)	20-100	-1.41	2.93
**HS 5**	143 (26)	67.3 (64.1-70.5)	19.2	70.0 (52.0-82.0)	21-100	-0.30	-0.72
**Total**	543 (100)	75.0 (73.5-76.5)	17.5	80.0 (65.0-90.0)	10-100	-0.81	0.31
