# Supplementary figures and images for: Health-related quality of life in breast cancer measured with EQ-5D-5L
Source: J Patient Rep Outcomes. 2026 Mar 20;10:67. doi: 10.1186/s41687-026-01044-x (PMC13125455; doi:10.1186/s41687-026-01044-x)

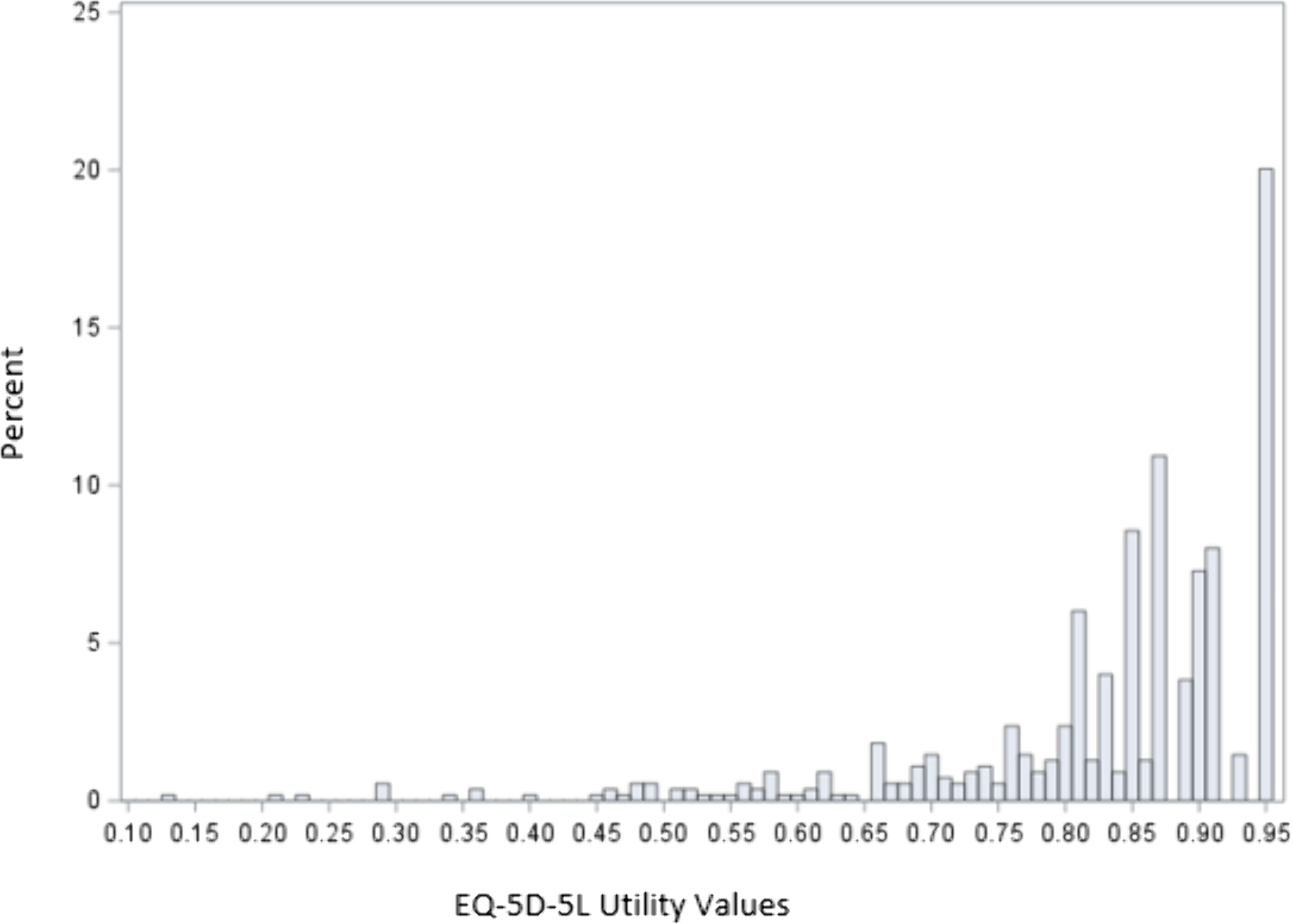

Supplement: Supplementary file 5 — Additional File 5.tif- Histogram for EQ-5D-5L health utility values [file 41687_2026_1044_MOESM5_ESM.tif]

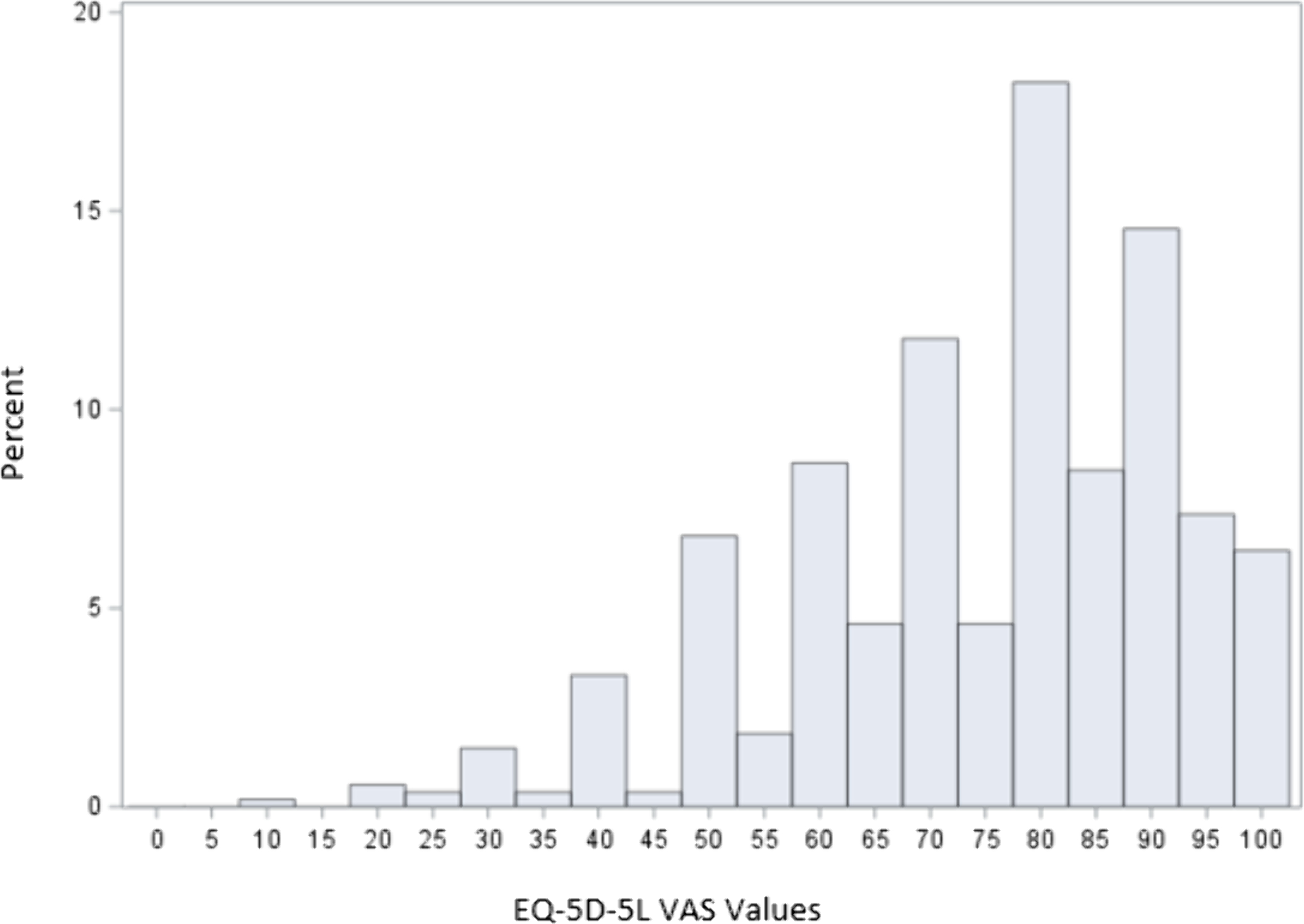

Supplement: Supplementary file 6 — Additional File 6.tif- Histogram for EQ-5D-5L VAS values [file 41687_2026_1044_MOESM6_ESM.tif]
